# A cultural training for the improvement of cognitive and affective Theory of Mind in people with Multiple Sclerosis: a pilot randomized controlled study

**DOI:** 10.3389/fpsyg.2023.1198018

**Published:** 2023-08-23

**Authors:** Alessia d’Arma, Annalisa Valle, Davide Massaro, Gisella Baglio, Sara Isernia, Sonia Di Tella, Marco Rovaris, Francesca Baglio, Antonella Marchetti

**Affiliations:** ^1^Unità di Urologia, Ospedale San Raffaele (IRCCS), Milan, Italy; ^2^Unità di Ricerca sulla Teoria della Mente, Dipartimento di Psicologia, Università Cattolica del Sacro Cuore, Milan, Italy; ^3^Fondazione Don Carlo Gnocchi Onlus (IRCCS), Milan, Italy; ^4^Università Cattolica del Sacro Cuore, Milan, Italy

**Keywords:** Multiple Sclerosis, rehabilitation, rehabilitation training, ToM, social cognition

## Abstract

Theory of Mind (ToM), the ability to understand and attribute mental states to ourselves and others, could be impaired in Multiple Sclerosis (MS), a neurodegenerative disease affecting young adults. Considering that ToM is strictly connected to Quality of Life (QoL) in MS and that could enhance the social support network -which is particularly important for this population-, we aimed to design and implement a novel ToM rehabilitation training. To make the training as much ecological as possible, we have devised a protocol enhancing ToM through stimuli depicting real-world conditions (video-clips taken from cinema movies, literary fictions, and audio voices). We test training’s effect on both cognitive and affective components of ToM in a sample of 13 subjects, randomly assigned to the ToM training Group and to the Control Group. The following ToM tasks were administered: the Reading the Mind in the Eyes (RMET), the Strange Stories task, the Faux Pas Task and the False Belief First- and Second - Order Task (FB II and III order). We also administered a psycho-behavioral assessment through the Toronto Alexithymia Scale (TAS-20). Results show that our novel ToM training is useful in enhancing ToM abilities measured by the following tasks: the RMET (affective task, *p* = 0.015) and the FB II-order task (FB, cognitive task, *p* = 0.032). Our ToM training had also a significant effect on the total score of the TAS-20 Scale (*p* = 0.018) and on its “Difficulty Describing Feelings subscale” (*p* = 0.018), indicating a reduction of the alexithymia traits. Future works with larger samples could investigate the ToM training effectiveness in a more representative MS populations.

## 1. Introduction

Successful participation in the social world requires a good comprehension of others’ thoughts, beliefs, and intentions, making accurate inferences about the contents of other people’s minds, and representing them in one’s mind. This complex process is made possible by Theory of Mind (ToM), the ability to understand and attribute mental states to ourselves and others (Premack and Woodruff, 1978), a foundational component of social cognition. ToM is developed throughout our entire life, but some clinical conditions or disturbances could have an impact on the development or maintenance of this ability. Among these, Multiple Sclerosis (MS), a chronic neurodegenerative disorder that impacts both the physical and cognitive status of people affected (pwMS). Recent literature has shown that pwMS show decreased performance in ToM tasks, both in its cognitive and affective domain (Raimo et al., 2017). Isernia et al. (2019) and Roca et al. (2014) suggest that only cognitive ToM is impaired in MS, while Cotter et al. (2016) highlighted a mainly affective ToM deficit in pwMS. Although there is still no agreement on which dimension is more involved, ToM deficit has a relevant impact on the life of pwMS, due to the strong association with reduced social and psychological quality of life (Giazkoulidou et al., 2019). For example, Bora et al. (2016) suppose that relational difficulties identified in pwMS could be related to the ToM deficit (Chiaravalloti and DeLuca, 2008). Considering that social support has an essential role in managing a disease that affects people at a young age (Costa et al., 2017), the treatment of ToM deficits for preserving interpersonal relationships in MS should be relevant in taking charge of this chronic condition. Considering the availability of a wide array of interventions targeted to the empowerment of ToM in other clinical conditions (Kurtz et al., 2016; Ashman et al., 2017; Edel et al., 2017; d’Arma et al., 2020) and in normal aging (featured by a decrease of ToM; Cavallini et al., 2015; Rosi et al., 2016), no training is currently established for the pwMS. For this reason, the present study aimed to design and preliminarily test the efficacy of a novel cultural ToM training (CToM; d’Arma et al., 2021) to promote emotional and cognitive mental state understanding in pwMS.

## 2. Materials and method

### 2.1. Participants

We conducted a RCT pilot study (Clinical Trial ID: NCT04711941, record nr: 5_25/07/2019); the randomization was computer-based type. A sample of 13 subjects with MS was consecutively recruited from the Neuromotor Rehabilitation Unit of Don Carlo Gnocchi Foundation, IRCCS in Milan [Italy; 6 participants were included in the experimental condition –ToM training – and 7 participants were included in the control condition (Non Mentalistic training)].

Inclusion criteria were: (1) diagnosis of Relapsing–Remitting (RR), Secondary Progressive (SP) and Primary Progressive (PP) MS based on the revised MC Donald criteria (Thompson et al., 2018), (2) age ≥ 18 and ≤75 years, (3) years of education ≥5, (4) stable pharmacological treatment in the 6 months before the enrollment, (5) no clinical relapses or use of steroid treatment during 3 months before the enrollment, (6) provided informed consent for study participation.

Exclusion criteria were: (1) history of nervous system disorders different from MS, (2) absence of unstable psychiatric illness, such as psychosis or major depression, (3) severe disability based on an Expanded Disability Status Scale (EDSS) score > 7.5, (4) severe cognitive impairment that would not enable pwMS to answer to questionnaires correctly (i.e., dementia), according to the patient’s medical records.

The study was conducted in compliance with the Helsinki Declaration of 1975, as revised in 2008. Local Ethics Committee (Don Carlo Gnocchi Foundation) approved the study, and written informed consent to be included in the study was obtained from participants before study initiation.

### 2.2. Neuropsychological, ToM, and psycho-behavioural assessment

Participants were randomly assigned to one of the two groups: the ToM training (experimental condition) and a non-mentalistic training (control condition).

Subjects belonging to both groups were individually assessed at the baseline (T0) and after 4 weeks at the end of the training (T1) regarding a neuropsychological evaluation, a ToM evaluation, and a psycho-behavioral evaluation. Blind neuropsychologists carried out the evaluation session. It was divided into two sessions, lasting about 1 h each.

Participants underwent a conventional neuropsychological, ToM and psycho-behavioral assessment as detailed in the Table 1 below.

**Table 1:** Assessment tasks.

Domain of evaluation	Test	Description
Neuropsychological	Montreal Cognitive Assessment (MoCA, Conti et al., 2015)	Global cognitive level
	Brief Repeatable Battery of Neuropsychological Test (BRB-NT; Rao, 1990).	Cognitive functions typically affected by MS
	Tests: Selecting Reminding Test Long Term Storage - SRT-LTS	Immediate verbal recall
	Selective Reminding Test Consistent Long-Term Retrieval - SRT-CLTR	Verbal memory storage
	Delayed Recall of the Selective Reminding Test - SRT-D	Verbal delayed memory
	Symbol Digit Modalities Test - SDMT	Visuospatial memory
	Paced Auditory Serial Addition Test - PASAT 2/ PASAT 3	Attention and processing speed
	Delayed Recall of the Spatial Recall Test - SPART-D	Visuospatial delayed recall
	Word List Generation - WLG	Access to lexicon through a semantic category
ToM task	Reading the Mind from the Eyes (RMET, Baron-Cohen et al., 2001)	Affective ToM
	Strange Stories (SS, Happé, 1994; Mazzola and Camaioni, 2002; Liverta Sempio et al., 2005)	Advanced ToM (mainly cognitive)
	Faux pas (Baron-Cohen et al., 1999)	Cognitive and affective advanced ToM
	False Belief Task (II and III order)	Cognitive ToM
Psycho-Behavioral assessment	Toronto Alexithymia Scale (TAS-20, Bagby et al., 1994)	Alexithymia measure

Demographical data were also collected by a neurologist: clinical onset, disease duration, EDSS score.

### 2.3. Procedures

After the baseline assessment, subjects were randomly assigned with the block randomization method to the experimental condition or to the control condition, each consisting of six 45-60 min lessons twice a week (for a total of 3 weeks). Training was set up during the ward-rehabilitation period of each PwMS. A psychologist conducted the training program in both conditions. The design of the two trainings was oriented by an ecological approach (Bechi et al., 2012, 2013; Cavallini et al., 2015), using stimuli derived from cinema and literature, since there is strong evidence that they could greatly impact ToM abilities (Mar et al., 2006, 2009; Fong et al., 2013; Kidd and Castano, 2013; Tabullo et al., 2018).

The Control training (Ct) activities were matched with those of the CToM for the modalities of delivery and type of material (number of lessons, length, visual stimuli, audio stimuli, and written stimuli). In the control condition, discussions in the lessons concerned physical and non-mentalistic states.

A full description of the training materials is provided in Table 2.

**Table 2:** Description of the ToMt and Ct.

		Experimental condition	Control condition
	Material	CToM	Ct
Lesson 1	Introduction and exemplification of the activities with practical exercises	Presentation of training aims, of the nature of the mentalistic attributions and of their importance in our daily lives. Practical exercises on “perspective taking” and on first and second false belief order. Reading of stories. Questions about characters’ mental states, beliefs and points of view.	Presentation of training aims; subjects were introduced to several existing media and communication/narrative languages. A descriptive overview of narrative (poetry, narrative fiction, media -radio, newscasts, newspapers, advertising) and not narrative (figurative arts, painting, photography) contents was proposed. Discussion on the material proposed.
Lesson 2	Audio stimuli	Listening to voices depicting emotions and intentions. Discussion with the trainer aimed to the correct interpretation and attribution of them.	Listening to podcasts of the most popular Radio program. Presentation of the narrative language proposed by this media. Focus on different techniques used to structure radio programs. Discussion on the technical aspects addressed by the lesson.
Lesson 3	Audio-visual Stimuli	Same activity proposed in Lesson 3 with different films.	Vision of video-clips of TV news and documentary taken from YouTube. Presentation of the narrative language proposed by these media. Focus on different techniques used to structure TV news report and documentary. Discussion on the technical aspects addressed by the lesson.
Lesson 4	Audio-visual Stimuli	Vision of video-clips of films taken from YouTube depicting social interactions. Questions about characters mental states (cognitive and affective), beliefs and points of view and discussion with the trainer.	Presentation of an historical overview of cinematographic language. Focus on the development of the cinematic language over the years with editing, camera movements and other cinematic techniques contributing specific roles in the narrative of films. Vision of video-clips from YouTube and discussion on the cinema technical aspects addressed by the lesson.
Lesson 5	Written stimuli	Reading of short narratives depicting social interactions. Questions about characters mental states (cognitive and affective), beliefs and points of view and discussion with the trainer.	Reading of a newspaper. Focus on the structure of a Typical Newspaper and on the characteristics of the potential readers target. Discussion on the technical aspects addressed by the lesson.
Lesson 6	Written stimuli	Same activity proposed in Lesson 5 with different short narratives.	Presentation of the language of advertising. Focus on types of advertising, the structure of advertising, the various types of clients with which advertising contents might interact. Discussion on the technical aspects addressed by the lesson.

#### 2.3.1. Cultural theory of mind training

For the design of the CToM presented in this study, we have considered existing literature: for the modalities (number of sessions, length, type of stimuli, etc.), we took into consideration the results of our previous systematic review and meta-analysis (d’Arma et al., 2020) and other ToM interventions already implemented in other clinical and not clinical population (Bechi et al., 2012, 2013, 2015; Cavallini et al., 2015; Lecce et al., 2015; Rosi et al., 2016). To respond to the need for an “ecological” treatment, we chose to use video, and audio stimuli depicting social interactions from cinema movies and TV fiction, as already implemented in Bechi et al. (2012, 2015). We proposed different activities (reading of stories, listening to audio voices, seeing a video-tape depicting human social interactions) to work on ToM capacity from different perspectives. The consideration of different skills that could contribute to cognitive or affective ToM could maximize the training’s impact on this ability. To make the training even more engaging, we also chose to use readings from fiction and literature. This idea is supported by a strand of work of Kidd and Castano (2013), Mar et al. (2006, 2009), Fong et al. (2013), and Tabullo et al. (2018), which have explored how literary fiction could enhance social abilities as empathy and ToM. This choice is supported by the results of an acceptability study confirming that CToM had been judged as very acceptable by pwMS (d’Arma et al., 2021).

During each training session, through several types of stimuli, the psychologist worked together with the participant to comprehend and hypothesize interpretations of the emotions and social interactions referred to the stimulus proposed (for example, scenes from movies or short stories), providing occasions in which participants were actively involved in the discussion in order to enhance the attribution of mental states and emotions. All materials proposed included exercises by which participants could answer to questions about thoughts and feelings of the characters; feedbacks from the psychologist conductor have served to raise the discussion about ToM contents.

#### 2.3.2. Control training

To compare our experimental condition with an active control group, we designed a parallel intervention not focused on ToM. According to the results emerging from our previous acceptability study (d’Arma et al., 2021), in which most pwMS reported making frequent use of communications media like newspapers, Tv and Radio, etc., we decided to design a pathway to deepen the different ways of communication, analyzing several types of existing communication. We provided a historical and descriptive overview of cinema movies, TV news, documentaries, newspapers, and advertising in every lesson. Particular attention was paid to the avoidance of mental states and emotional reasoning during the presentation of the stimuli and the subsequent discussion. The proposal’s objective was to avoid enhancing the ToM, mindreading competencies, and executive functions. As in the experimental condition, participants were actively involved in the discussion: All materials proposed included exercises by which participants could answer to questions about descriptive features of the contents proposed. The aim of this condition was to maintain the same format of the experimental condition with the clause of not taking in consideration any issues relating to thoughts and feelings (to avoid the bias of indirectly ehanching ToM).

### 2.4. Statistical analysis

The analyses were performed with SPSS 24.0. and with Jamovi statistical software (The Jamovi Project 2020, version 1.2).

For the description of the sample’s demographic characteristics, descriptive analyses were performed, including means, standard deviation (SD), and range for continuous variables as appropriate.

To test if and how our CToM could impact ToM abilities, a statistical comparison between experimental subjects and control participants was performed.

Before evaluating the effect of the training, we compared at the baseline the two groups to assess if they were comparable through the ANOVA one way method. For the demographic variables, we confirmed the normal distribution with the Shapiro–Wilk normality test.

To analyze the CToM effect, we conducted an analysis of the variance with a mixed model with a random effect. The analysis was performed through a linear mixed model fit by REML algorithm. As Fixed Effect parameters we put time, group, age, and education. As random components, subjects and residual are set. Convergence is present in all the tests performed with mixed model.

## 3. Results

Table 3 reports the principal demographic characteristics of the participants and the group’s comparison. One subject was considered a drop out because of its exit from the study (the subject had to discontinue its hospitalization for the first Italian lockdown on March 2020).

**Table 3:** Demographic, ToM and neuropsychological characteristics of the participants.

Variables	ToM training (*N* = 6)	Non-mentalistic training (*N* = 7)	*p* value
Sex (M/F)	2/4	3/4	0.725^#^
Age (years) [average ± SD (min – max)]	61 ± 9.92 (47–75)	60 ± 14.30 (39–74)	0.749*
Education (years) [average ± SD (min – max)]	12.33 ± 3.67 (8–17)	10.86 ± 2.67 (6–7.5)	0.352*
MS phase (RR/SP/PP)	2/2/2	3/4/0	0.452*
Disease duration (years) [average ± SD (min – max)]	22.5 ± 16.56 (2–39)	25.43 ± 8.56 (12–36)	0.503*
EDSS score [average ± SD (min – max)]	6.33 ± 0.26 (6–6.5)	6.14 ± 0.90 (6–7.5)	0.703*

N, number; M, male; F, female; MS, Multiple Sclerosis; Min, Minimum; Max, Maximum; RR, Relapsing Remitting; SP, Secondary Progressive; PP, Primary Progressive; EDSS, Expanded Disability Status Scale; MoCA, Montreal Cognitive Assessment.

# χ^2^ Tests.

* ANOVA One Way.

The two groups did not differ significantly for any of the demographic variables considered; hence they are comparable. The age, the education, and the disease duration were normally distributed, except for the EDSS score.

In Table 4 results of the analysis of the variance were shown.

**Table 4:** Analysis of variance with a linear mixed model.

Task	Time	CToM (average ± SD)	Ct (average ± SD)	Range/Cut Off	*p* value (Time × Group)	*F* value (Time × Group)	*p* value after Bonferroni correction
MoCA test	T0	23.83 ± 2.56	23.50 ± 4.08	26	0.217	1.73	0.409
	T1	25.00 ± 1.41	22.50 ± 3.45				
Strange Stories (ToM)	T0	18.83 ± 3.71	13.00 ± 3.79	0–24	0.122	2.89	0.310
	T1	20.50 ± 2.43	17 ± 4.65				
Strange Stories (Physical)	T0	3.5 ± 0.55	3.5 ± 0.55	0–8	0.642	2.8482	0.837
	T1	3.67 ± 0.82	3.5 ± 1.22				
RMET	T0	22.66 ± 5.39	18.50 ± 5.57	0–36	**0.015**	8.65	**0.038**
	T1	25.16 ± 5.41	16.33 ± 6.62				
False Belief II order	T0	2.50 ± 0.55	3.00 ± 1.41	0–5	**0.032**	5.23	0.061
	T1	4.50 ± 0.84	3.00 1.41				
False Belief III order	T0	4.20 ± 1.94	3.66 ± 1.37	0–8	0.115	2.87	0.060
	T1	3.67 ± 2.16	1.67 ± 1.33				
Faux Pas (ToM)	T0	19.33 ± 7.53	20.83 ± 8.30	0–30	0.115	5.16	0.066
	T1	22.50 ± 8.17	13.33 ± 7.45				
Faux Pas (Physical)	T0	8.00 ± 4.00	8.67 ± 1.63	0–10	0.352	0.95	0.522
	T1	7.67 ± 4.08	7.3 ± 2.42				
Faux Pas Control questions (ToM)	T0	9.33 ± 1.21	9.83 ± 0.41	0–10	0.935	0.01	0.246
	T1	9.50 ± 0.84	10.00 ± 0				
Faux Pas Control Questions (Physical)	T0	10.00 ± 0.00	10.00 ± 0.00	0–10	0.345	0.94	0.110
	T1	9.33 ± 0.82	9.67 ± 0.52				
SRT-LTS	T0	41.33 ± 7.58	34.17 ± 12.58	23.3	0.514	0.46	0.342
	T1	42.50 ± 7.92	32.3 ± 18.70				
SRT-CLTR	T0	33.17 ± 4.92	27.17 ± 15.22	15.5	0.274	1.34	0.339
	T1	33.50 ± 7.71	22.67 15.38				
SPART	T0	19.83 ± 4.26	19.33 ± 6.74	12.7	0.813	0.06	0.930
	T1	17.50 ± 3.73	16.67 ± 5.09				
SDMT	T0	42.33 ± 7.09	37.83 ± 8.82	37.9	0.153	2.38	0.333
	T1	37.33 ± 6.02	38.30 ± 13.11				
PASAT 3	T0	35.67 ± 7.39	31.00 ± 1.63	28.4	0.060	4.51	0.140
	T1	29.17 ± 10.46	32.50 ± 10.37				
PASAT 2	T0	26.33 ± 4.03	19.33 ± 5.85	17.1	0.895	0.01	0.472
	T1	26.50 ± 7.74	20.00 ± 5.66				
SRT-D	T0	8.17 ± 0.75	6.50 ± 2.07	4.9	0.748	0.11	0.611
	T1	7.17 ± 1.84	6.0 0 ± 3.03				
SPART-D	T0	5.67 ± 0.82	6.50 ± 1.97	3.6	0.058	4.51	0.367
	T1	7.17 ± 1.17	6.00 ± 1.67				
WLG	T0	23.83 ± 6.85	19.33 ± 6.22	17	**0.048**	5.08	0.433
	T1	20.00 ± 6.07	20.17 ± 6.43				
TAS – Total Score	T0	52.83 ± 12.22	57.67 ± 12.75	0–100	**0.018**	3.06	0.073
	T1	43.33 ± 11.64	59.00 ± 18.23				
TAS – Identification Subscale	T0	18.83 ± 6.14	17.50 ± 5.09	25	0.501	0.49	0.773
	T1	14.67 ± 7.34	15.84 ± 8.42				
TAS – Description Subscale	T0	15.67 ± 4.55	13.67 ± 6.12	35	**0.018**	7.99	0.122
	T1	10.67 ± 3.83	15.67 ± 6.12				
TAS – Think Esternally Oriented Subscale	T0	18.33 ± 5.95	26.5 ± 4.28	40	0.702	0.15	**0.004**
	T1	18.00 ± 3.79	27.50 ± 5.75				

MoCA, MOntreal Cognitive Assessment; RMET, Reading the mind in the eyes test; ToM, Theory of Mind; SRT-LTS, Selecting Reminding Test Long Term Storage; SRT-CLTR, Selective Reminding Test Consistent Long-Term Retrieval; SPART, Delayed Recall of the Spatial Recall Test; SDMT, Symbol Digit Modalities Test; PASAT, Paced Auditory Serial Addition Test; SRT-D, Delayed Recall of the Selective Reminding Test; SPART-D, Delayed Recall of the Spatial Recall Test; WLG, Word List Generation; TAS, Toronto Alexithymia Scale.

**Figure 1:**
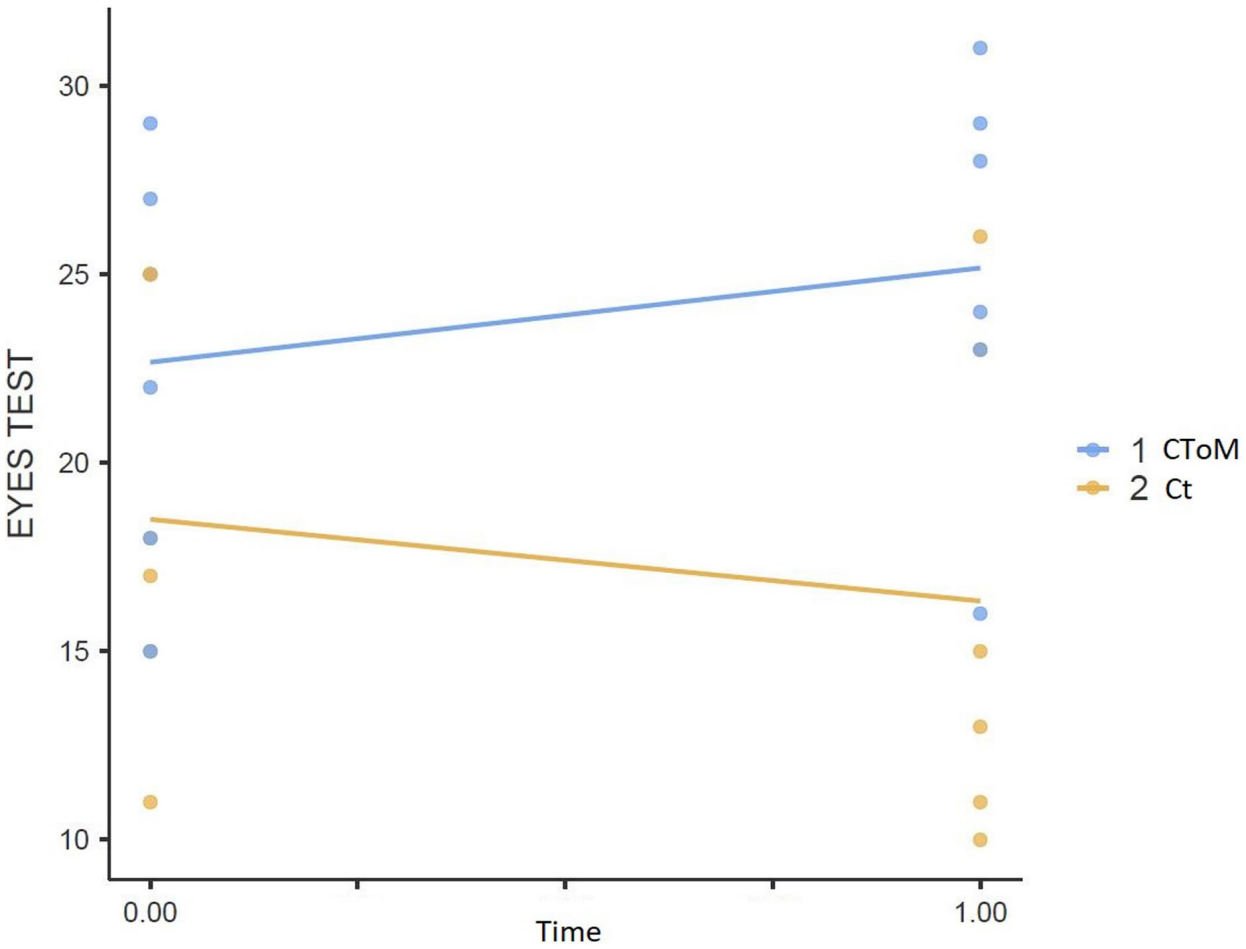
Eyes test result pre vs. post.

**Figure 2:**
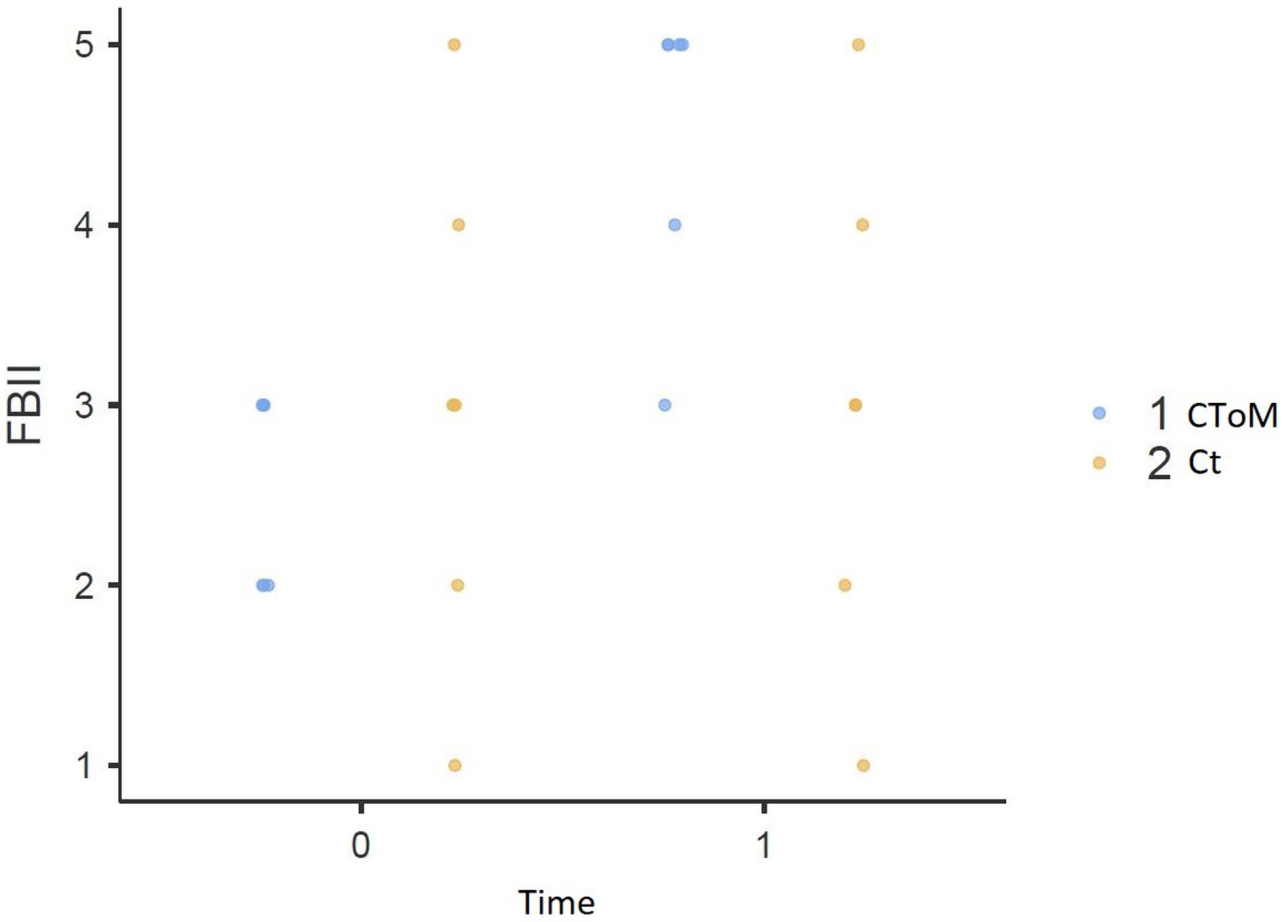
False belief task result pre vs. post.

**Figure 3:**
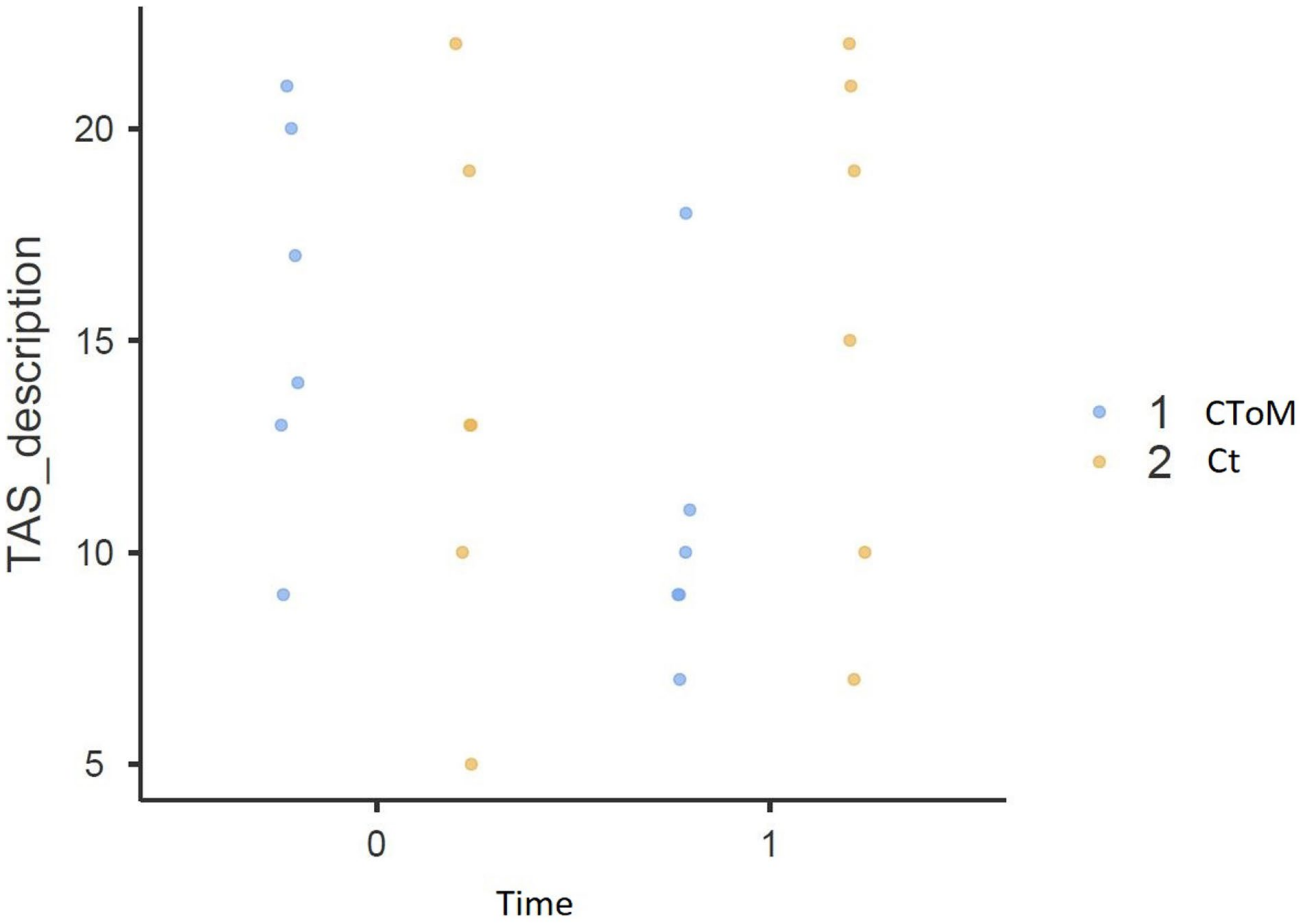
Toronto Alexithymia Scale decription result pre vs. post.

**Figure 4:**
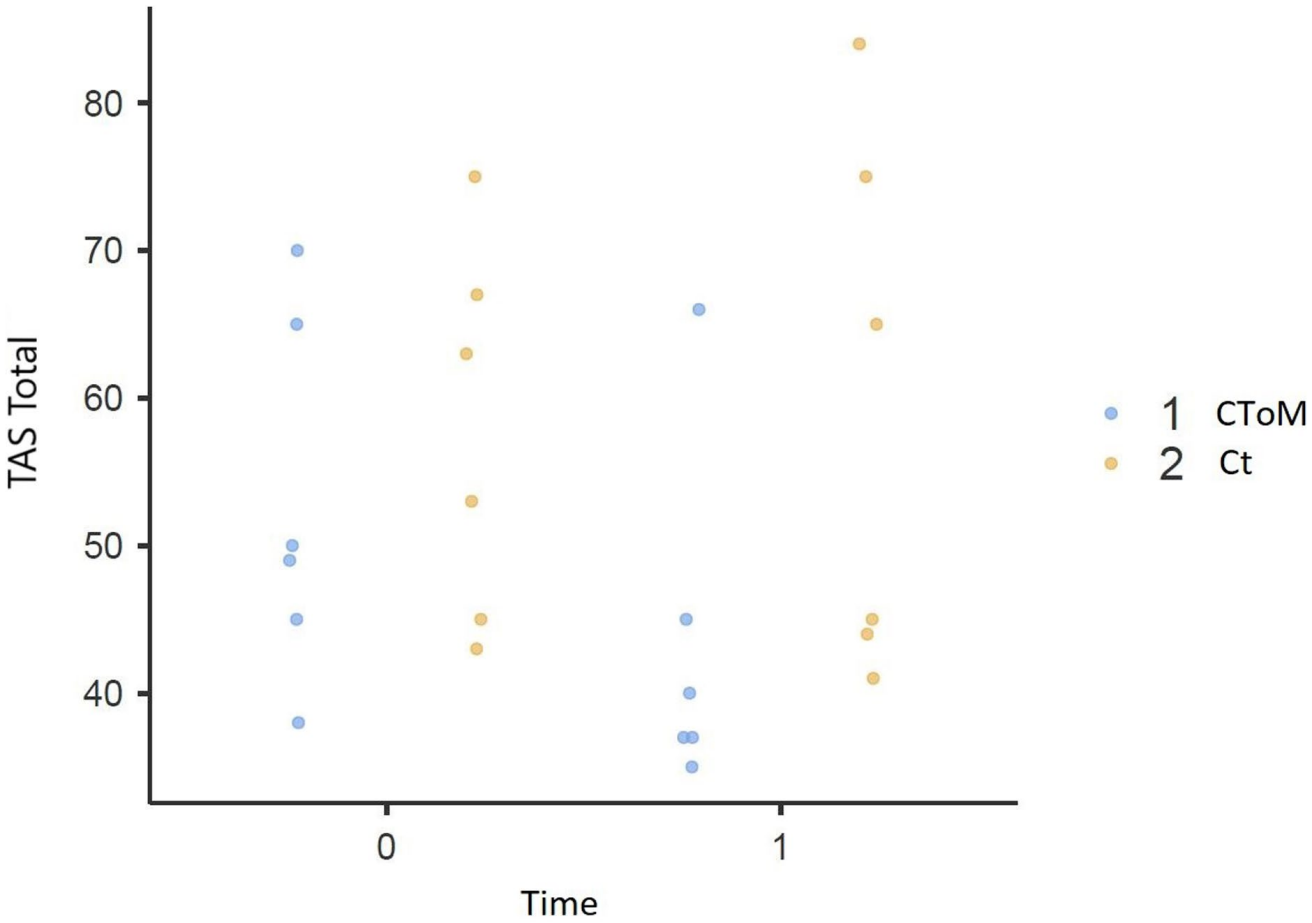
Toronto Alexithymia Scale total score result pre vs. post.

**Figure 5:**
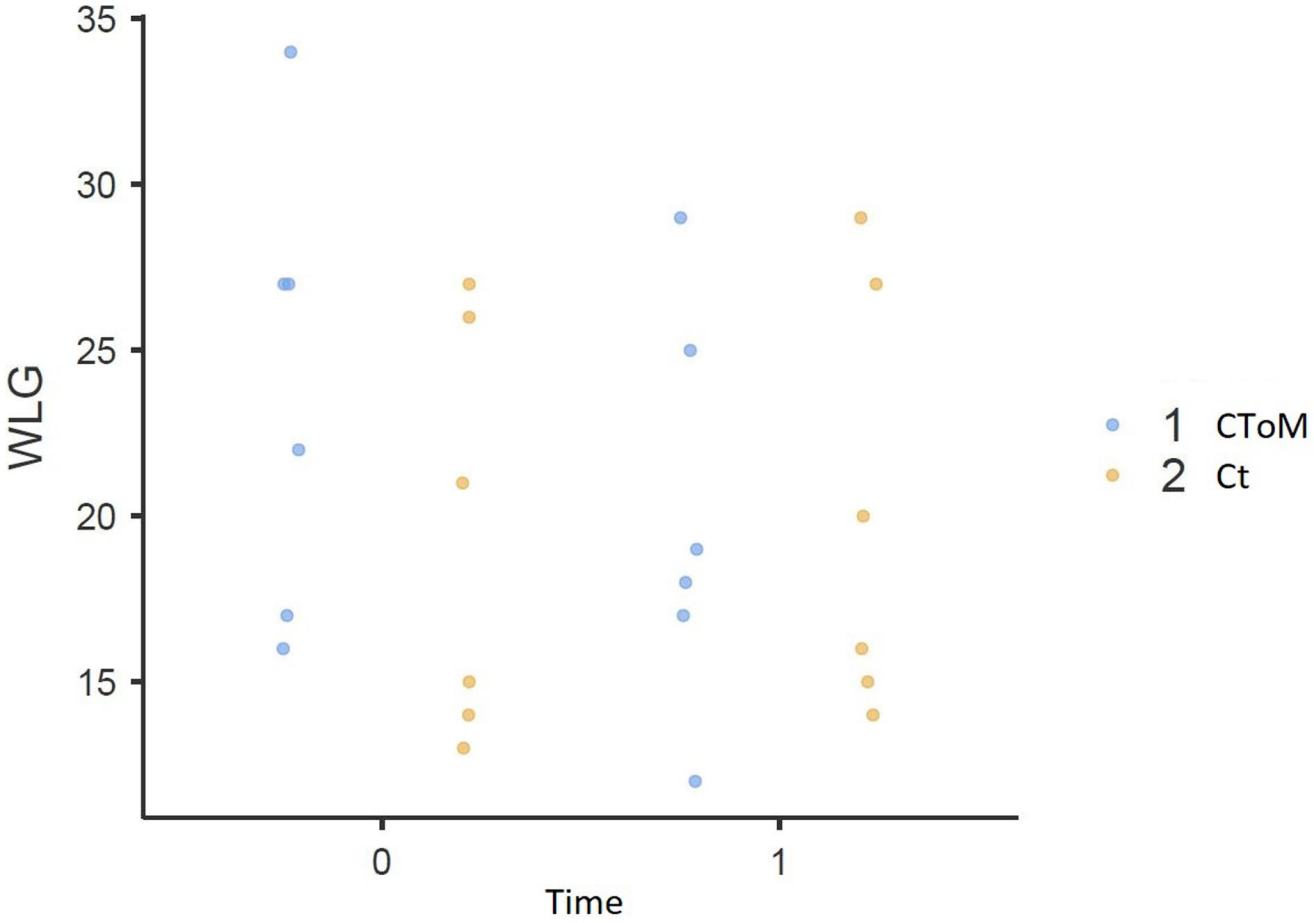
Word generation list result pre vs. post..

We found a statistically significant Time × Group effect on the RMET test (*p* = 0.015), on the False Belief II order task (*p* = 0.032), on the WLG test (*p* = 0.048), in the total score of the TAS-20 (*p* = 0.018) and the Description Subscale of the TAS-20 (*p* = 0.018) (Figures 1–5).

## 4. Discussion

These results of our study show that our novel CToM training could be useful in the enhancement of ToM, in both affective and cognitive components. Regarding the affective ToM, the analysis showed a highly significant effect of the training on the RMET; the use of cultural stimuli improves ToM and particularly the affective component, as shown in the work of Kidd and Castano (2013). Specifically, our CToM training enhances the emotion recognition of the characters of the cultural stimuli presented (deriving from cinema and literature), contextualizing them in the several conditions displayed. We can assume that fictions (read or seen) propose narratives and frame significant for the subjects (Bruner and Bruner, 1990), motivating them to apply their ToM ability. To understand the stimuli of the CToM training, mainly important is to correctly attribute the emotions to the characters; this training is composed by situations in which the protagonists experience different emotions in the face of the same event and, as in real-life, the adequate interpretation of emotional states guide the understanding of the situation itself.

Regarding the cognitive ToM, our CToM training has also shown a significant effect on the second-order false-belief task, but not on the third-order reasoning. This is probably since that higher ToM reasoning is difficult to do even for non-clinical people (see, for example, Valle et al., 2015), then it is probably inaccessible in a clinical situation characterized by a ToM deficit. Instead, the second-order ToM, usually reached around 8 years of age (Perner and Wimmer, 1985), can be more sensitive to the CToM, that proposes multiple situations regarding, for example, gaffes or non-direct communications, requiring second-order recursive reasoning to be understood. Nevertheless, the CToM training does not seem to generalize its impact on the advanced ToM tasks, the Strange Stories, and the Faux pas test, for which we found a trend toward significance. This is probably due to the complexity of the advanced ToM tasks, that requires very high competences in the social context, that pwMS could not reach due to the MS disease. Furthermore, a very small number of the subjects have been included in this study: in the future, a sample’s enlargement could show a more evident impact on ToM abilities.

These results support the idea that a generic training rehabilitation, such as the Ct here proposed, is not enough to enhance the specific ability of ToM in a sample of clinical population: on the contrary, it seems that is precisely the work on the comprehension of emotions and mental states together with the enhancement of mental states inferences to be effective in enhancing basic ToM abilities (as happen also in other type of population, i.e., primary school children; Bianco et al., 2021). This result is corroborated by the fact that there was no statistically significant improvement in both groups in the neuropsychological evaluation.

Only in the WLG task, assessing verbal fluency, the control condition shows a higher performance at T1 respect to the T0. This effect could be due to the nature of the control condition: indeed, even though we pose particular attention to avoid the focus on mentalistic ability in the non-mentalistic training, the six control condition lessons were characterized by lengthy discussions on the different types of communication currently available. Although non-mentalistic training seems to be not effective on ToM domain and other executive functions, it is possible that the fluency could be enhanced by the discussion held in the control condition.

Our CToM also had a significant effect on the alexithymia, reduced in the experimental group, indicating an improvement of the alexithymia traits after the CToM. Alexithymia was a relevant issue in patients with MS (Eboni et al., 2018), and it was associated with increased severity of fatigue and depression (Bodini et al., 2008). The result obtained in the “Difficulty Describing Feelings subscale” of the TAS-20 in favor of the experimental group confirms that conversations and discussions about mental states and emotions in an ecological manner could help pwMS describe feelings and emotions. Because one of the problems in managing MS is the improvement of the patient-physician interaction (Alroughani, 2015), this result confirms the importance to set ToM trainings in order to translate the results from the research setting to clinical practice, overcoming the gap between research discoveries and routine practice of treatments (Rothwell, 2005).

There are several limits to be considered for the interpretation of these results. The Covid-19 pandemic emergency negatively impacted the recruitment of the subjects: subjects were recruited during their rehabilitation hospitalization in the Rehabilitation Unit of the Multiple Sclerosis Center of the Don Gnocchi Foundation that, for several months, were stopped to give priority to another kind of hospitalization most appropriate at that stage. So, at that moment we have been able to recruit only 13 subjects, of which one has been considered a drop out just due to anti-COVID 19 strategic measures (the subject had to discontinue its hospitalization for the first Italian lockdown on March 2020). To date, the rehabilitation unit is still open and resumed MS rehabilitation hospitalization, however not without some inevitable changes. Operators must wear several personal safety protection devices that could impact the relationship between the training and the inpatients even if they do not interfere with the training itself. Related to this limit, we have to pinpoint that we are aware of the limit in fitting a linear mixed model despite the small number of our populations; this is only a pilot study and we had the need to understand the effect of our ToM training controlling contents and methods of the treatment, in order to give an indication of differences among times × groups.

Furthermore, pwMS recruited from our Rehabilitation Unit is often in a stage of disease with moderate and severe disability, making it difficult to generalize these preliminary results to the MS population, which presents a greater degree of variability.

## 5. Conclusion

Our study provides preliminary findings on the efficacy of our CToM training for rehabilitating and enhancing the ToM abilities in pwMS. The effect is detectable not only on ToM tasks (both affective and cognitive), but also on alexithymia traits. Due to the crucial repercussions that they have on social interactions, social support networks, and QoL, the possibility to implement rehabilitation training for the empowerment of ToM ability in this population should be considered in clinical settings. However future studies are needed for verifying the effectiveness of four rehabilitation methods in samples with larger size and more representative of the MS population of patients.

## Data availability statement

The original contributions presented in the study are included in the article/supplementary material, further inquiries can be directed to the corresponding author.

## Ethics statement

The studies involving human participants were reviewed and approved by Comitato Etico - Fondazione Don Carlo Gnocchi. The patients/participants provided their written informed consent to participate in this study.

## Author contributions

AV, FB, DM, and AM conceived and planned research and contributed to the management, the coordination of the research activity, the supervision of the evolution of the research goals and of the revision of the manuscript. Ad'A conceived and planned research, collected data and contributed to the data entry, to statistical analysis, to the synthesis and the presentation of study data, drafting and submitting the manuscript. GB contributed to data collection and data entry. SI contributed to data collection and revision of the manuscript. MR managed and cohordinated the patient recruitment and contributed to the revision of the manuscript. Ad'A and AV wrote the first draft of the manuscript. SDT that perform statistical analysis, revised the manuscript and contribute to the final form of the manuscript. All authors contributed to the article and approved the submitted version.

## Funding

This work was funded by the Italian Ministry of Health (“Ricerca Corrente” - 2022-2024 Program).

## Conflict of interest

The authors declare that the research was conducted in the absence of any commercial or financial relationships that could be construed as a potential conflict of interest.

## Publisher’s note

All claims expressed in this article are solely those of the authors and do not necessarily represent those of their affiliated organizations, or those of the publisher, the editors and the reviewers. Any product that may be evaluated in this article, or claim that may be made by its manufacturer, is not guaranteed or endorsed by the publisher.
